# Substantial Remission in Subacute Sclerosing Panencephalitis by Following the Ketogenic Diet: A Case Report

**DOI:** 10.7759/cureus.5485

**Published:** 2019-08-25

**Authors:** Janak Nathan, Dhanashri Khedekar Kale, Vidula D Naik, Forum Thakker, Sonal Bailur

**Affiliations:** 1 Neurology, Shushrusha Hospital, Mumbai, IND; 2 Clinical Nutrition & Dietetics, Dr. Nathan Sanjiv Clinic, Mumbai, IND

**Keywords:** subacute sclerosing panencephalitis, epilepsy, ketogenic diet

## Abstract

An eight-year-old boy presented with rhythmic myoclonic jerks that stretched back to the age of four years. He was diagnosed as having subacute sclerosing panencephalitis (SSPE). This is a progressive and almost uniformly fatal disease. His condition gradually deteriorated till he was unable to speak or walk. He also experienced incontinence and severe cognitive decline (stage 3a in the Risk and Haddad scale). An electroencephalogram (EEG) showed myoclonic jerks with periodic, generalised, high-amplitude and slow-wave complexes. Cerebrospinal fluid (CSF) findings also were supportive of the diagnosis of SSPE. The ketogenic diet (KD) therapy was started on the patient. His myoclonic jerks stopped after 11 months. After 36 months, his cognition and physical abilities vastly improved. His EEG showed no slow-wave complexes and background activity was almost normal.

SSPE is secondary to measles and causes inflammatory and neurodegenerative changes. KD has an anti-inflammatory effect and can halt and reverse neurodegenerative changes. Its neuroprotective effects could be due to the reduced oxidative stress, enhanced mitochondrial activity, and the suppression of pro-apoptotic factors. Thus, KD could control the myoclonic jerks and also reverse the cognitive and physical decline arising from SSPE.

## Introduction

Subacute sclerosing panencephalitis (SSPE) is a chronic, progressive, inflammatory, and degenerative affliction of the brain caused by a persistent infection by, or, mutation of the measles virus (MV) [1]. SSPE primarily affects children and young adults, with the estimated rate of infections ranging from one in 10,000 to one in 609 persons infected with MV [1-2]. Serological studies have reported a high annual incidence (21 cases per one million population) of SSPE in India [3]. Mortality usually occurs within one to three years from the onset of the disease [3]. The pathogenesis of SSPE is related to direct infection of the central nervous system (CNS) by MV, causing progressive inflammation and sclerosis (neurodegeneration) of the brain due to an impaired immune response [4-5]. The spread of MV in the CNS is not through the extracellular virus but could be trans-synaptic [4]. Apoptosis of various cell types may contribute to the neuropathogenesis of the MV infection in the CNS, either as a direct effect of viral infection or of cytokine-mediated responses, resulting in the neuronal cell death in SSPE [4]. Persistent MV infection may be due to inflammatory factors as evidenced by the high MV antibodies and the presence of MV throughout the body [4]. The usual incubation period of SSPE following measles infection is six to eight years [4]. It is probably caused by the mutant MV’s ability to spread from cell to cell while evading cell-mediated immunity in the brain [4]. The ketogenic diet (KD) has been demonstrated to be effective in drug-resistant epilepsy [6]. It also has a positive effect on the immune response in addition to its anti-seizure, anti-inflammatory effects and neuroprotective action. It is also found to halt and reverse neurodegeneration [6-7]. All of these factors could play a role in the alleviation and control of SSPE. There is one case report of its use in SSPE, which describes only a temporary cessation of the myoclonic jerks and finds no effect on the physical or cognitive parameters [8]. We describe a case of SSPE treated with KD that led to a significant improvement in the physical parameters and cognition and resulted in the cessation of the myoclonic jerks.

## Case presentation

**Figure 1:**
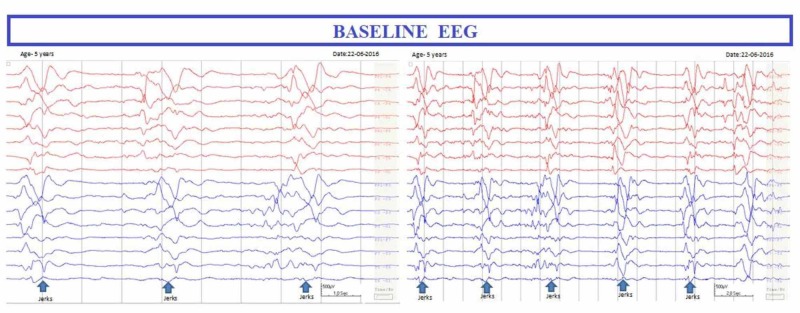
Baseline EEG. Solid arrows denote the clinical myoclonic jerks that are accompanied by high-amplitude and stereotyped generalised slow-wave complexes.

An eight-year-old boy, not immunised against measles, had clinical measles at two years of age. Two years later, he presented with myoclonic jerks and repeated falls. His electroencephalogram (EEG) showed myoclonic jerks with typical fairly stereotyped and periodic, generalised, high-amplitude and slow-wave complexes diagnostic of SSPE (Figure 1).

He was on three antiviral medications (isoprinosine 500 mg/day, ribavirin 100 mg/day, and lamivudine 100 mg/day). He was also on four anti-seizure medications (sodium valproate 200 mg/day, clonazepam 0.5 mg/day, levetiracetam 250 mg/day, and clobazam 5 mg/day). However, there was no reduction in the frequency or intensity of the myoclonic jerks. His physical and mental condition continued to deteriorate rapidly. He presented to us in a moribund state (Risk and Haddad stage 3a) but was still able to take feeds orally. All medications had been stopped.

The cerebrospinal fluid quotient reference (CSFQ ref) is the ratio between the cerebrospinal fluid (CSF)/serum measles-specific immunoglobulin G (IgG) quotient and the CSF/serum total IgG quotient. A CSFQ ref of more than 1.5 is considered to be indicative of measles-specific antibody production in the CNS. His CSFQ ref was 3.02, and his anti-measles IgG antibody was found to be 399 (positive >275) IU/I. Four oligoclonal bands (OCBs) were detected in the CSF, but they were absent in the serum. Both of these phenomena corroborated the possibility of a measles etiology and were also indicative of intrathecal IgG synthesis (Table 1).

**Table 1:** Cerebrospinal Fluid (CSF) Serology Chart. Abbreviations: CSF – cerebrospinal fluid; IgG – immunoglobulin G.

Date	Serum IgG Measles U/ml	CSF IgG Measles U/ml	Serum Total IgG mg/dl	CSF Total IgG mg/dl	Relative CSF/Serum Quotient	CSF Protein mg/dl	Anti Measles-IgG Antibody IU/I
16.12.2015	13412.5	15051.6	1360	8.08	0.93 (Normal)	38.5	--
31.03.2018	12156.5	15185.6	1560	13.1	3.02 (Positive >1.5)	34.8	399.0 (Positive >200 )

His baseline and follow-up biochemical parameters were normal. A brain MRI showed T2 and fluid-attenuated inversion recovery hyperintensity in the corona radiata of bilateral frontoparietal regions extending into the periventricular region, with diffuse cerebral atrophy and dilatation of the lateral and third ventricles.

Due to the failure of the conventional treatment, he was put on KD as a monotherapy. Pre-KD, his height was 102 cm and weight was 11.9 (ideal body weight (IBW): 17.2 kg). KD was started at a ketogenic ratio (the ratio of fat to carbohydrate plus protein in grams) of 2:1, with 100% recommended dietary caloric allowance to achieve the IBW and later fine-tuned to maintain the IBW and urine ketone of 4+ (160 mg/dL) throughout the day. His blood ketone level was maintained at an average of 2.2 mmol/L. Vitamins, minerals, and a urine alkalinizing agent were prescribed.

**Figure 2:**
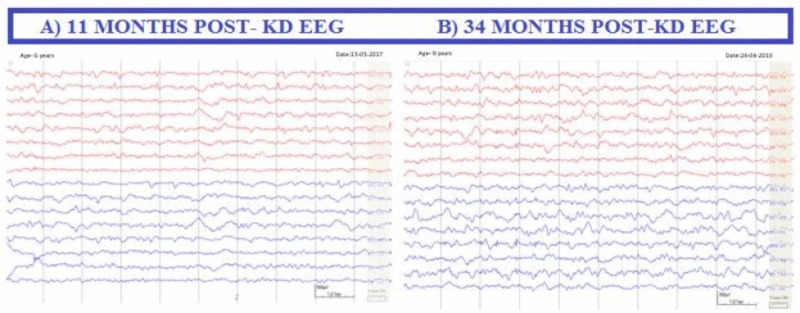
Post-ketogenic diet EEG. Shows a mildly slow background activity. No generalised slow-wave complexes are seen, and clinically no myoclonic jerks are seen.

Within one month, his ability to maintain eye contact returned. His myoclonic jerks ceased completely after 11 months. He now sits without support and can stand for a few seconds. He has now started speaking in monosyllables and begun to show emotions such as smiling and crying. He is still continuing with KD and shows continuous, though gradual, improvement. His EEG now shows no rhythmic and stereotyped generalised slow-wave complexes and background activity is almost normal (Figure 2).

There were no side effects seen except for occasional nausea and vomiting. Compliance and tolerability were excellent as ascertained by a diet diary and a daily record of urine ketones.

## Discussion

Our patient had SSPE as per Dyken’s criteria, with two major criteria (clinical history and CSF findings) and one minor criterion (typical EEG) [9] present. He showed marked improvement in his cognition and physical abilities and complete control of jerks while on KD, resulting in improved well-being. On KD, there was a rise in his CSFQ ref (3.02) and presence of OCBs in the CSF, which supports the diagnosis of a CNS measles infection and ongoing IgG activation in the brain (Table 1). The Risk and Haddad study categorises six stages and several sub-stages of SSPE based on the severity of symptoms [10]. Our patient was at 3a stage, namely, vegetative psychomotor condition [10]. Spontaneous remission has been reported in stage 4 of the Risk and Haddad scale in between 53% to 10% of cases [10]. Spontaneous remission may occur during any stage of the disease and last for a variable period before an eventual relapse occurs [11].

Antiviral drugs and immunomodulators are commonly used in the treatment of SSPE [11]. Antiviral drugs interfere with the viral replication by preventing or inhibiting one of the key components necessary for viral assembly [11]. The altered characteristics of the mutant MV in SSPE may by itself hamper the effectiveness of these antiviral drugs [11]. The combined use of both isoprinosine and interferon alpha was found to have some initial effectiveness but not in the long term [12]. In our patient, steady deterioration was noted despite the use of antiviral agents and anti-epileptic medications.

Neuronal fibrillary tangles (NFTs), which are associated with altered levels of tau and related proteins in CSF, have been observed in certain SSPE cases [11]. NFTs act by inducing glucose hypometabolism and reducing mitochondrial function due to increased superoxide production with oxidative damage and thus alter brain metabolic activity [11].

There are possibly multifactorial mechanisms which could explain the action of KD in SSPE. Ketones increase the cerebral after-discharge and suppress the expression of pro-apoptotic factors, which correspond to enhanced rates of recovery from seizure episodes [6]. They may also improve cell-survival capacity and increase the hippocampal expression of the calcium-binding protein, calbindin, and thus limit neuronal hyperexcitability induced by seizures [6]. This could be helpful in controlling the myoclonic jerks. However, seizure control by itself cannot fully explain the improvement in physical and cognitive skills. Ketones have an anti-inflammatory effect by increasing the nicotinamide adenine dinucleotide (NAD+/NADH) ratio, which may influence gene expression through Sirtuin 1 (SIRT 1) activation and limit oxidative stress by improving synthesis of heat shock proteins, increasing antioxidant defences and promoting DNA repair activity [6-7]. They decrease the production of reactive oxygen species (ROS) by complex I of the mitochondrial respiratory chain and improve the mitochondrial function by increasing metabolic efficiency [7]. They also stimulate the cellular endogenous antioxidant system by activating the major inducer of detoxification genes, nuclear factor (erythroid-derived 2)-like-2 (Nrf2) [7]. Ketones provide an efficient alternative source of energy to ameliorate the glucose hypometabolism caused by NFTs [7]. Ketones activate SIRT 1, which deacetylases NFTs by targeting ubiquitination and proteosomal cleavage and reducing tangles [7]. Ketones increase autophagy and reduce levels of hyperphophorylated NFTs deposition in the hippocampus, amygdala, and cortex in Alzheimer’s patients [13]. Ketones also upregulate the brain-derived neurotrophic factor through the nuclear factor kappa-B as an adaptive response against ROS, mediating neuroprotection against NFTs [7]. Being synapse builders, ketones can improve synaptic plasticity and protect against neuronal damage caused by NFTs [7]. They can also exert neuroprotective action by activating the energy-sensing signalling pathways [7]. The above anti-seizure, anti-inflammatory, and neuroprotective effects of ketones and their ability to halt neurodegeneration could be responsible for the improvement in this case of SSPE, though in varying degrees.

As mentioned before, spontaneous remission can be seen in SSPE patients with long-term remission being rare [9-11]. At present, there is a paucity of human studies on the use of KD in SSPE. The only reported case of the use of KD in a patient with SSPE showed that the myoclonic jerks temporarily subsided but reappeared and were then refractory to the treatment [8]. The effect seen here of the KD is promising, and it suggests that KD is a useful adjunct in the treatment of SSPE. This is particularly worthwhile in view of the resurgence of the disease across the world due to reduced vaccination rates. A longer follow-up period, as well as a multicentre, randomised controlled trial, will be required for affirming the effectiveness of KD in SSPE. We hope this case report will provide the impetus for such a trial.

## Conclusions

KD has a beneficial role in the management of SSPE and may be effective even as a monotherapy. We believe this is the first case where a therapy has been shown to be successful in stopping and reversing the progress of SSPE. The patient is continuing with KD and is progressively improving. A multicentre, randomised controlled trial will be helpful in determining the potential of the KD therapy in SSPE.
